# Plaster of Paris

**Published:** 1905-05

**Authors:** Wm. H. Gelston


### PLASTER OF PARIS.[1]

BY WM. H. GELSTON, D.D.S.

Plaster of Paris, so named because of the situation of the original beds under the city of Paris, is an anhydrous calcium sulphate, which before being calcined is a hydrated sulphate of lime, which is undoubtedly derived from the precipitation of gypsum by evaporation from its solution in circumscribed basins of salt water, like the Dead Sea and Great Salt Lake. Gypsum is also usually associated with greater or less quantities of the salts which are found in sea-water,—namely, the chlorides of sodium, calcium, magnesium, the sulphate of soda, etc. Of all the solid matter contained in sea-water, gypsum is the least soluble, and, therefore, is the first precipitated.

In the carboniferous age evaporating pans where salt water precipitated its solid content existed in Nova Scotia, Michigan,

[1] Read before the Southern Dental Society of New Jersey, December 21, 1904.

Virginia, and Arizona. The samples I pass you were taken from the Province of New Brunswick on the Bay of Fundy, where the finest gypsum is quarried to-day. These are all practically pure, containing about ninety-nine per cent. sulphate of lime; they came from various veins or strata in the quarry, and my object in exhibiting them is to show how a sulphate of lime can vary. Being soluble in water it is not infrequent that a pond or lake is to be found in the vicinity of plaster quarries, in the bottom of which is a deposit of sulphate of lime in crystals. The late Professor Cope, of the Academy of Natural Sciences of Philadelphia, contended that the marl-beds of New Jersey, extending from, say, near old White Horse (Berlin) toward Perth Amboy, contained more or less sulphate of lime, and after an exhaustive search he succeeded in finding a number of beautiful and perfectly formed crystals, some of which are deposited in the Academy of Natural Sciences of Philadelphia as convincing proof of his theory, and one of which I have much pleasure in submitting for your examination. I also have one taken from the original beds under the city of Paris. These have been kindly loaned me by Mr. French. And possibly it might not be out of place to state that I am indebted to Mr. French (the president of the College of Pharmacy and senior member of the firm of Samuel H. French & Co., who manufacture almost the entire output of plaster for the dental profession) for his time and courtesies extended in giving samples and statistics that so materially aided in my presentation of this paper.

Gypsum is not only an indispensable article in the form of plaster, but is used extensively by the two most famous breweries of Dublin and London, tons being annually dumped into their wells, wherein lies the success of these famous brews.

Let us spend just a moment at the factory. Gypsum as quarried is thrown into large breakers, which crush it into pieces varying in size from a small pea to a large chestnut. This in turn is carried by conveyors to mills that grind it to an exceedingly fine powder; this powder is conveyed to kettles, where it is calcined, or, to use a commercial term, and one that is familiar to all calciners, where it is "boiled." These kettles have a capacity of about one hundred barrels each. The surplus moisture is thrown off, which causes the plaster to have the appearance of boiling, bubbles forming on the top during the process and breaking and emitting steam. This boiling process causes a material expansion, owing to the generation of steam. After the moisture has all been driven off, the plaster rapidly settles, and to a novice it would almost appear as though the bottom of the kettle had dropped out, but in reality it is simply returning to its original bulk in minute crystal formations. At this stage the heat is not only continued, but is increased so as to break the small crystals and drive off the water of crystallization. After this has been accomplished the calcining process is completed. It is scarcely necessary to say that in calcining plaster it requires a technical knowledge only attained by long experience.

I have given you simply an outline of the calcining process, and it is, of course, to be understood that, in addition to this process in making dental plaster, there are mechanical and technical processes used which have been kept as a manufacturing secret and which is possessed by but few. In other words, the processes by which the setting of plaster is hastened or retarded for the use of the profession is a manufacturing secret.

I will state, in addition to my remarks on calcining plaster, that, from information which I have, I am convinced that the mechanical processes used in the production of dental plaster absolutely prevent any variation in quality in a batch of from nine hundred to twelve hundred barrels. In other words, if one barrel is wrongly manufactured, it must necessarily follow that from nine hundred to twelve hundred barrels are in the same condition, and this would be detected, as each batch is thoroughly tested before leaving the factory.

The dental plaster as manufactured by Samuel H. French & Co. is in three varieties,—impression, setting in from three to five minutes; dental, in from eight to ten minutes; slow-setting, in from thirty to forty minutes. This firm also manufactures an exceedingly slow-setting plaster, which, however, they do not produce for the use of the profession, but for that of the arts.

We will now take up, if you please, the textile strength and different forms of plaster relative to the amount of expansion. The textile strength of plaster is as follows: Twenty-four hour mix, one hundred and fifty-three pounds to the square inch; seven day mix, two hundred and eighty-four pounds to the square inch; twenty-eight day mix, two hundred and ninety-four pounds to the square inch; six months mix, three hundred and fifteen pounds to the square inch; one year mix, three hundred and seventy-two pounds to the square inch.

EXPANSION.

In all these experiments careful relative measurements and temperature of plaster and water were taken before mixing; afterwards, time of stirring, setting, and expansion.

Impression plaster, seven months old, expanded as high as one-sixteenth of an inch, while new expanded $^1/_{136}$.

The expansion of impression plaster with boiled water and boiled lime-water was exactly the same,—$^1/_{100}$.

A saturated solution of plaster of Paris in boiled water before mixing, expansion, $^1/_{300}$.

Impression plaster, boiled water and salt, expansion $^1/_{136}$.

Impression plaster mixed very thin expanded $^1/_{136}$.

Impression plaster with boiled snow expanded $^1/_{136}$.

Impression plaster with cold hydrant water 38° expanded $^1/_{200}$.

Impression plaster with melted snow at 38°, no expansion in twenty hours.

Impression plaster with melted snow at 38°, stirred thirty seconds instead of five, expansion $^1/_{100}$.

Dental plaster in the experiments was about the same as impression.

Slow-setting plaster and boiled water, expansion $^1/_{300}$, after forty-eight hours $^1/_{200}$.

Sixty-minute or gelatin plaster with boiled water, expansion none in three days, after thirty days, expansion $^1/_{200}$.

From the results of these experiments the following might be suggested: Plaster should not be purchased in quantities sufficient to become old before using, as old plaster expands more than new. It should be kept well covered and away from dampness as much as possible. Mr. French states that complaints are confined principally to fall and spring, which seems due to the fact that the heavy rains of both seasons infiltrate the water with foreign matter. Use at least boiled water for impressions and models. Less expansion with a thin mix than with a thick one. Rain water preferable to hydrant. Water at about 36° lessens expansion. Allow the plaster to slack in water rather than long stirring, which latter promotes expansion. Less expansion in slow-setting plaster than either dental or impression plaster. Always use sixty-minute plaster where possible for models. A model will remain harder during the process of vulcanizing if not vulcanized the same day as poured. The steam should be blown off as soon as the case is vulcanized, and the flask cooled to prevent disintegration of the plaster. Subjecting impression plaster to different temperatures of heat, care being taken not to scorch it, produces a slower setting plaster, and if carried to a high degree a plaster can be produced that will require eighty-five minutes to harden with no expansion in twenty-four hours.

After these and many more experiments the subject would seem to be more expansive than the plaster itself, which has as many eccentricities as a bucking broncho, and I know whereof I speak, for I have been " up against" both. But possibly it might be a little more accurate if I used the statement of Mr. French, that " it is as peculiar as individuals." And if you will stop to consider the individual as you meet him day after day in the office, you will all agree some are harder to manage than a broncho.

### DISCUSSION.

Dr. Chas. Tuttle claimed that he secured very good results in mixing plaster for models by using boiled water. No reason was given why this should be preferred to the unboiled water.

Dr. J. B. McCullough, gave his method (previously published in the INTERNATIONAL DENTAL JOURNAL) of overcoming the expansion of plaster, by the use of a solution of lime-water, made from water and lime boiled for fifteen minutes; then mixed with the plaster.

Dr. W. W. Crate stated that his method of use, after mixing plaster, was to prepare the plate from the model, as soon as possible for vulcanization.

*Dr. Gelston.*—In answer to Dr. McCullough, I do not consider his tests with glass tubes accurate or conclusive. I used glass tubes with some of my first experiments with ordinary hydrant water, and some of them cracked while others did not; this being the case, I found it necessary to abandon that method for a more accurate measurement. By actual measurement, I received no better results from boiled lime water than from boiled water. But you will notice from some of the experiments that I mentioned, where I did receive better results than from either boiled water or lime water.

